# Human T-cell lymphotropic virus type-1 infection associated with sarcopenia: community-based cross-sectional study in Goto, Japan

**DOI:** 10.18632/aging.103736

**Published:** 2020-07-24

**Authors:** Hirotomo Yamanashi, Kenichi Nobusue, Fumiaki Nonaka, Yukiko Honda, Yuji Shimizu, Shin-Ya Kawashiri, Mai Izumida, Yoshinao Kubo, Mami Tamai, Yasuhiro Nagata, Katsunori Yanagihara, Bharati Kulkarni, Sanjay Kinra, Atsushi Kawakami, Takahiro Maeda

**Affiliations:** 1 Department of General Medicine, Nagasaki University Graduate School of Biomedical Sciences, Sakamoto, Nagasaki 852-8501, Japan; 2 Department of Infectious Diseases, Nagasaki University Hospital, Sakamoto, Nagasaki 852-8501, Japan; 3 Department of Clinical Medicine, Institute of Tropical Medicine, Nagasaki University, Sakamoto, Nagasaki 852-8523, Japan; 4 Department of Island and Community Medicine, Nagasaki University Graduate School of Biomedical Sciences, Goto, Nagasaki 853-8691, Japan; 5 Department of Community Medicine, Nagasaki University Graduate School of Biomedical Sciences, Sakamoto, Nagasaki 852-8523, Japan; 6 Department of Immunology and Rheumatology, Nagasaki University Graduate School of Biomedical Sciences, Sakamoto, Nagasaki 852-8523, Japan; 7 Department of Innovative Development of Human Resources for Comprehensive Community Care, Nagasaki University Graduate School of Biomedical Sciences, Sakamoto, Nagasaki 852-8523, Japan; 8 Department of Laboratory Medicine, Nagasaki University Graduate School of Biomedical Sciences, Sakamoto, Nagasaki 852-8523, Japan; 9 Clinical Division, National Institute of Nutrition, Hyderabad 500007, India; 10 Department of Non-communicable Disease Epidemiology, London School of Hygiene and Tropical Medicine, London WC1E 7HT, UK

**Keywords:** sarcopenia, HTLV-1, chronic inflammation, appendicular skeletal muscle mass, handgrip strength

## Abstract

Sarcopenia is characterized by a progressive skeletal muscle disorder that involves the loss of muscle mass and low muscle strength, which contributes to increased adverse outcomes. Few studies have investigated the association between chronic infection and sarcopenia. This study aimed to examine the association between human T-cell lymphotropic virus type-1 (HTLV-1) and sarcopenia. We conducted a cross-sectional study and enrolled 2,811 participants aged ≥ 40 years from a prospective cohort study in Japanese community dwellers during 2017–2019. Sarcopenia was defined as low appendicular skeletal muscle mass and low handgrip strength. The association between HTLV-1 seropositivity and sarcopenia was assessed using multivariable logistic regression. Odds ratio (OR) and 95% confidence interval (CI) of sarcopenia were analysed using HTLV-1 seropositivity. We adjusted for age, sex, body mass index, physical activity, systolic blood pressure, glycated haemoglobin, low-density lipoprotein cholesterol, and smoking and drinking status. Of 2,811 participants, 484 (17.2%) HTLV-1 infected participants were detected. HTLV-1 infection was significantly associated with sarcopenia (adjusted OR 1.46, 95% CI 1.03–2.07, P = 0.034). HTLV-1 was associated with sarcopenia among community-dwelling adults. Active surveillance and early detection of asymptomatic HTLV-1 infection might be beneficial to reinforce countermeasures to inhibit the progress of HTLV infection-associated sarcopenia.

## INTRODUCTION

Sarcopenia is characterized by a progressive and generalized skeletal muscle disorder that involves the accelerated loss of muscle mass and low muscle strength [1]. Sarcopenia, a major problem associated with aging, is listed in the International Classification of Diseases-10 code [2], and is associated with increased adverse outcomes including falls, disability, hospital admission, long term care placement, poor quality of life, and mortality [3–5]. Estimates of disease frequency have varied, but became more precise with the evolution of its definition. One of the lowest pooled prevalence estimates (12.9% [95% confidence interval; CI 9.9–15.5]) was calculated using a 2010 definition of the European Working Group on Sarcopenia in Older People [1, 6]. Sarcopenia has a medical and economic burden; the total estimated cost of hospitalizations of individuals with sarcopenia in the USA was USD $40.4 billion with a mean per person cost of USD $260 [7].

Factors that cause and worsen sarcopenia are categorised as primary and secondary. Known risk factors include changes in sex steroid hormones and cytokines associated with aging, genetic susceptibility (as primary sarcopenia), physical inactivity, smoking, low 1,25-OH vitamin D, low nutritional intake, low protein intake, and atherosclerosis (as secondary sarcopenia) [6, 8–17].

A recent study reported that sarcopenia was more prevalent in a community cohort of human immunodeficiency virus (HIV) infection [18]. In the cross-sectional analysis of 315 HIV-infected cases and matched controls, sarcopenia was more prevalent in HIV-infected cases compared with matched controls [19]. Except for HIV infection, however, no study has investigated the association between chronic infection and sarcopenia.

Human T-cell lymphotropic virus type-1 (HTLV-1) was the first retrovirus shown to cause diseases including adult T-cell leukaemia, HTLV-1 associated myelopathy/tropic spastic paraparesis, polymyositis and dermatomyositis [20–22]. HTLV-1-infected CD4^+^ T-lymphocytes were found to infiltrate the muscle of patients with inflammatory myopathies, and cytotoxic T-lymphocyte immune reactions to the *TAX* gene induced tumour necrosis factor-alpha and cytotoxic effects on muscles [22]. However, little is known about the effect of HTLV-1 infection on muscles in asymptomatic carriers.

We hypothesized that chronic inflammation caused by HTLV-1 infection has a negative impact on skeletal muscle and strength. However, the relationship between HTLV-1 infection and sarcopenia has not been investigated to date. The aim of this study was to investigate whether there is an association between HTLV-1 infection and sarcopenia and to examine whether chronic inflammation is associated with sarcopenia.

## RESULTS

### General characteristics of the study population

Among 2,811 participants with a mean age of 70 years (standard deviation [SD] ± 10.0), 502 (18%) HTLV-1-infected participants were detected using a chemiluminescent enzyme immunoassay (CLEIA). Samples from 488 of these 502 participants were available for real-time PCR (RT-PCR) testing. Of these 474 were positive for HTLV-1 and 14 were negative by RT-PCR. For the negative cases, western blotting showed that three cases were positive, one was negative, one was indeterminate, and Innogenetics line immunoassay showed that seven cases were positive, one was indeterminate, and one was negative. The 484 (17%) participants who were positive for HTLV-1 by RT-PCR, western blotting or Innogenetics line immunoassay were included in the analysis.

Table 1 shows the characteristics of participants with HTLV-1 infection. Compared with HTLV-1-uninfected participants, HTLV-1-infected participants included a lower proportion of men (29.1% vs 39.8%, P < 0.001), and those with older age (mean age 74.0 vs 69.4, P < 0.001), lower weight (55.6 kg vs 57.8 kg, P < 0.001), lower handgrip strength (24.7 kg vs 28.4 kg, P < 0.001), lower appendicular skeletal muscle mass (ASM) (15.4 kg vs 16.5 kg, P < 0.001), and higher proportion of antihypertensive drug use (50.6% vs 40.7%, P < 0.001). The body mass index (BMI), proportion of hypoglycaemic drug use, lipid-lowering drug use, physical activity, systolic blood pressure (SBP), glycated haemoglobin (HbA1c), high-density lipoprotein cholesterol (HDL), triglycerides (TG), high sensitive C-reactive protein (hs-CRP), and white blood cells (WBC) were not different between HTLV-1-infected participants and HTLV-1-uninfected participants.

**Table 1:** Demographic and clinical characteristics of HTLV-1 infected and uninfected participants.

	**HTLV-1 uninfected Mean ± SD or N (%)**	**HTLV-1 infected Mean ± SD or N (%)**	**P-value**
N	2327 (82.8)	484 (17.2)	
Age (year)	69.4 ± 10.1	74.0 ± 8.2	<0.001 ^c^
Sex (male)	925 (39.8)	141 (29.1)	<0.001 ^d^
Height (cm)	156.7 ± 8.8	153.3 ± 8.2	<0.001 ^c^
Weight (kg)	57.8 ± 10.4	55.6 ± 9.8	<0.001 ^c^
Body mass index	23.4 ± 3.1	23.6 ± 3.3	0.604 ^c^
Handgrip strength (kg)	28.4 ± 9.6	24.7 ± 8.8	<0.001 ^c^
Appendicular skeletal muscle mass (kg)	16.5 ± 4.1	15.4 ± 3.7	<0.001 ^c^
Sarcopenia	143 (6.2)	59 (12.2)	<0.001 ^d^
Smoking status			<0.001 ^d^
Current	230 (9.9)	26 (5.4)	
Past	568 (24.4)	92 (19.0)	
Never	1529 (65.7)	366 (75.6)	
Drinking status			0.019 ^d^
Current	850 (36.5)	145 (30.0)	
Past	96 (4.1)	19 (3.9)	
Never	1381 (59.4)	320 (66.1)	
Antihypertensive drug use	946 (40.7)	245 (50.6)	<0.001 ^d^
Hypoglycemic drug use	157 (6.8)	37 (7.6)	0.478 ^d^
Lipid-lowering drug use	483 (20.8)	108 (22.3)	0.444 ^d^
Physical activity	1.23 ± 0.76	1.23 ± 0.77	0.889 ^d^
Systolic blood pressure (mmHg)	134.8 ± 18.1	135.7 ± 18.1	0.214 ^c^
Diastolic blood pressure (mmHg)	75.7 ± 11.0	74.3 ± 10.5	0.018 ^c^
Glycated haemoglobin (%)	5.76 ± 0.55	5.77 ± 0.51	0.147 ^c^
Low-density lipoprotein cholesterol (mg/dl)	122.2 ± 30.4	117.9 ± 29.9	0.014 ^c^
High-density lipoprotein cholesterol (mg/dl)	61.8 ± 15.2	60.4 ± 15.0	0.055 ^c^
Triglycerides (mg/dl)	105.2 ± 60.8	106.5 ± 65.1	0.595 ^c^
High sensitive C-reactive protein (μg/dl) ^a^	(191, 389, 827)	(190, 423, 923)	0.187 ^c^
White blood cells (/μl)^b^	(4500, 5300, 6300)	(4500, 5300, 6400)	0.503 ^c^

Data on high sensitive C-reactive protein and white blood cells were expressed as the median and interquartile range.

^a^ N=2712, ^b^ N=2804

^c^ P values from Wilcoxon rank sum test.

^d^ P values from McNemar chi-square test.

### Sarcopenia and HTLV-1 infection

In univariable linear regression analysis, HTLV-1 infection had a positive association with sarcopenia (ß = 0.09, P < 0.001) (Table 2). The standardized ß coefficient was positively significant for age, antihypertensive agent use, hypoglycaemic agent use, SBP and WBC. This coefficient was inversely significant for male sex, height, weight, BMI, smoking and drinking status, diastolic blood pressure and HDL.

**Table 2:** Univariable linear regression analysis for the effect of each exposure on handgrip strength, apendicular skeletal muscle mass and sarcopenia (N=2811).

**Variable**	**Handgrip strength**		**Appendicular skeletal muscle mass**		**Sarcopenia**	
	**ß**	**P-value**	**ß**	**P-value**	**ß**	**P-value**
HTLV-1 infection	-0.14	<0.001	-0.11	<0.001	0.09	<0.001
Age (year)	-0.38	<0.001	-0.24	<0.001	0.24	<0.001
Sex (male)	0.71	<0.001	0.63	<0.001	-0.04	0.041
Height (cm)	0.73	<0.001	0.74	<0.001	-0.22	<0.001
Weight (kg)	0.57	<0.001	0.68	<0.001	-0.22	<0.001
Body mass index	0.13	<0.001	0.27	<0.001	-0.14	<0.001
Smoking status	0.51	<0.001	0.45	<0.001	-0.05	0.013
Drinking status	0.42	<0.001	0.37	<0.001	-0.06	0.001
Antihypertensive agent use	-0.12	<0.001	-0.04	0.044	0.10	<0.001
Hypoglycemic agent use	-0.01	0.568	0.05	0.008	0.04	0.042
Lipid-lowing drugs use	-0.07	<0.001	-0.06	0.003	0.04	0.059
Physical activity	0.03	0.166	0.01	0.673	-0.03	0.067
Systolic blood pressure (mmHg)	-0.08	<0.001	-0.08	<0.001	0.06	0.001
Diastolic blood pressure (mmHg)	0.14	<0.001	0.09	<0.001	-0.04	0.045
Glycated haemoglobin (%)	-0.01	0.640	0.03	0.107	0.01	0.682
Low-density lipoprotein cholesterol (mg/dl)	-0.05	0.010	-0.07	<0.001	-0.03	0.102
High-density lipoprotein cholesterol (mg/dl)	-0.14	<0.001	-0.18	<0.001	-0.05	0.010
Triglycerides (mg/dl)	0.08	<0.001	0.07	0.001	-0.02	0.348
High sensitive C-reactive protein (μg/dl) ^a^	0.00	0.935	0.04	0.019	0.03	0.172
White blood cells (/μl) ^b^	0.10	<0.001	0.07	<0.001	0.05	0.009

^a^ N=2712, ^b^ N=2804

In logistic regression analysis, HTLV-1 infection was significantly associated with low handgrip strength (odds ratio [OR] 2.06, 95% CI 1.62–2.62, P < 0.001). This association remained significant after further adjustment for age, sex, BMI, physical activity, SBP, HbA1c, low-density lipoprotein cholesterol (LDL), and smoking and drinking status (adjusted OR [aOR] 1.35, 95% CI 1.04–1.76, P = 0.024) (Table 3). HTLV-1 infection was associated with low skeletal muscle mass index (SMI) (OR 1.24, CI 1.01–1.53, P = 0.040), but this association was not significant after further adjustment (aOR 1.11, 95% CI 0.87–1.40, P = 0.402). HTLV-1 infection was significantly associated with sarcopenia (OR 2.12, 95% CI 1.54–2.92, P < 0.001), and remained significant after further adjustment (aOR 1.46, 95% CI 1.03–2.07, P = 0.034).

**Table 3:** Adjusted odds ratio and 95% confidence interval for HTLV-1 infection in relation to sarcopenia (N=2811).

	**Low handgrip strength**			**Low appendicular skeletal muscle mass index**			**Sarcopenia**	
	**aOR**	**95% CI**	**P-value**	**aOR**	**95% CI**	**P-value**	**aOR**	**95% CI**	**P-value**
Model 1	1.38	(1.06, 1.79)	0.016	1.09	(0.86, 1.37)	0.473	1.48	(1.05, 2.09)	0.027
Model 2	1.35	(1.04, 1.76)	0.024	1.11	(0.87, 1.40)	0.402	1.46	(1.03, 2.07)	0.034

Model 1. Adjusted for age, sex and body mass index.

Model 2. Model 1 + physical activity, systolic blood pressure, glycated haemoglobin, low-density lipoprotein cholesterol, smoking and drinking status.

aOR: adjusted odds ratio, CI: confidence interval.

We also tested the association between HTLV-1 infection and sarcopenia among participants who were excluded as premenopausal women to avoid pre/postmenopausal changes to muscle mass and strength. The aOR did not differ after excluding premenopausal women (aOR 1.46, 95% CI 1.03–2.06, P = 0.035).

### Sarcopenia and inflammatory markers

Logistic regression analysis was performed to examine the association between sarcopenia and inflammatory markers (logarithm of hs-CRP and WBC). Hs-CRP was significantly associated with low SMI after adjustment for age, sex, BMI, physical activity, SBP, HbA1c, LDL, and smoking and drinking status (aOR 1.19, 95% CI 1.10–1.29, P < 0.001) (Table 4). WBC was significantly associated with low SMI and sarcopenia after adjustment (aOR 2.31, 95% CI 1.62–3.31, P < 0.001; aOR 3.26, 95% CI 1.77–5.99, P < 0.001, respectively).

**Table 4:** Adjusted odds ratio and 95% confidence interval for sarcopenia in relation to inflammatory markers.

**Variable**	**Model**	**Low handgrip strength**			**Low appendicular skeletal muscle mass index**			**Sarcopenia**		
		**aOR**	**95% CI**	**P-value**	**aOR**	**95% CI**	**P-value**	**aOR**	**95% CI**	**P-value**
High sensitive CRP (logarithm)^a^											
	Model 1	1.04	(0.94, 1.15)	0.450	1.19	(1.10, 1.29)	<0.001	1.12	(0.98, 1.27)	0.089
	Model 2	1.04	(0.94, 1.14)	0.490	1.19	(1.10, 1.29)	<0.001	1.10	(0.96, 1.25)	0.156
White blood cells (logarithm)^b^										
	Model 1	1.44	(0.93, 2.23)	0.104	2.32	(1.64, 3.29)	<0.001	3.37	(1.86, 6.11)	<0.001
	Model 2	1.46	(0.93, 2.29)	0.097	2.31	(1.62, 3.31)	<0.001	3.26	(1.77, 5.99)	<0.001

Model 1. Adjusted for age, sex and body mass index.

Model 2. Model 1 + physical activity, systolic blood pressure, glycated haemoglobin, low-density lipoprotein cholesterol, smoking and drinking status.

^a^ N=2712, ^b^ N=2804

aOR: adjusted odds ratio, CI: confidence interval.

## DISCUSSION

This community-based cross-sectional study demonstrated that asymptomatic HTLV-1 infection is a risk factor for sarcopenia. To the best of our knowledge, this is the first report to show chronic HTLV-1 infection is a risk factor for sarcopenia. In addition, inflammatory makers were identified as risk factors for low SMI and sarcopenia. Sarcopenia has recently been recognized as an emerging issue in HIV infection [18]. Multicenter AIDS cohort studies of over 7000 HIV patients’ in the USA reported that handgrip strength and walking speed declined more rapidly in HIV infected people compared with uninfected controls [23, 24]. In these studies, the authors suggested the possible mechanism of functional decline might have resulted from inflammation induced by co-infection with hepatitis C virus, diabetes mellitus, chronic kidney disease, or peripheral neuropathy. In a cross-sectional study in Malaysia, HIV-infected individuals had a higher proportion of sarcopenia defined by the Asian Working Group for Sarcopenia than HIV-uninfected individuals [17% (n = 8) vs 4% (n = 2), P = 0.049] [19]. In that study, the authors suggested that higher sarcopenia susceptibility in HIV-infected individuals was associated with chronic immune activation and inflammation. Contrary to symptomatic HIV infection under antiretroviral therapy, participants of our study were asymptomatic carriers of HTLV-1 infection and were not treated with any viral therapy. Therefore, there was no bias related to drug-induced modification of the association between HTLV-1 infection and sarcopenia in our study. Furthermore, our results suggested that HTLV-1 infection might have a cumulative risk for sarcopenia, even in the asymptomatic phase.

A putative mechanism of HTLV-1 associated sarcopenia is warranted. Potential explanations include anabolic resistance induced by inflammation and triggered catabolism. Because HTLV-1 promotes the production of cytokines such as interferon-gamma, tumour necrosis factor-alpha, and interleukin-6 [25], through the activation of nuclear factor-κB and cyclic AMP response element-binding protein [26], inflammatory factors might be induced even during asymptomatic infection. CRP and WBC are well-known surrogate markers of infection, and are activated by interleukin-6 or tumour necrosis factor-alpha produced by monocytes or macrophages. Inflammation contributes to the impaired mammalian/mechanistic target of rapamycin signalling (anabolic resistance), which leads to insufficient muscle protein synthesis [27]. Furthermore, inflammatory cytokines also promote protein catabolism [28].

Another putative pathway of HTLV-1 associated sarcopenia is an overabundance of reactive oxygen species (ROS). HTLV-1 encodes viral structural genes of the pX region encoding *TAX*, which increases the production of ROS [29, 30]. ROS, chemically unstable reactive free radicals that cause endothelial dysfunction, were reported as risk factors for sarcopenia [31]. ROS contribute to age-related deficits in muscle through increased oxidative damage to cell constituents and/or through the induction of defective redox signalling. ROS induced oxidative stress also damages other cellular components such as DNA, proteins, and lipids resulting in further damage to the cells and tissues. Consequently, the intra and intercellular membranes of the muscle fibres, in particular those of the sarcoplasmic reticulum, may be modified and the Ca2^+^ transport mechanism altered [32].

In a retrospective study, thirteen French HTLV-1 infected-patients with polymyositis or dermatomyositis showed moderate muscle inflammation compared with HTLV-1 infected-controls without myopathy. However, the level of the proviral load did not differ between these groups [22]. Although a high proviral load is a major risk factor for adult T-cell leukaemia and HTLV-1 associated myelopathy/tropic spastic paraparesis [33], level of proviral load was not a determinant of HTLV-1 infection inducing muscle inflammation [22]. Therefore, even though HTLV-1 infected study participants were asymptomatic carriers, HTLV-1 infection may lead to subclinical muscle inflammatory reaction and sarcopenia.

The associations between HTLV-1 infection and low handgrip strength, and HTLV-1 infection and sarcopenia were significant, but not between HTLV-1 infection and low SMI. These inconsistent results may be partially explained by the loss of muscle strength, which is typically clinically apparent before the loss of muscle mass. Data from a longitudinal study in Baltimore showed that a decline in muscle strength was much greater than that predicted by the decline in muscle mass [32].

Some limitations of the present study should be mentioned. First, as this was a cross-sectional study, we were not able to establish cause–effect relationships. Second, we had no data on the infectious period of each participant. Because effects of HTLV-1 infection on sarcopenia progression are thought to be cumulative, differences in the duration of infection may bias the results. Third, although the study participants were recruited from healthy community dwellers, we cannot deny the mixture of HTLV-1 infected-patients with polymyositis or dermatomyositis. However, although the association between HTLV-1 infection and sarcopenia was not significant, the same tendency was observed after excluding participants with joint pain (n = 639), which is a common symptom of inflammatory myopathy (aOR 1.47, 95% CI 0.98–2.21, P = 0.062) [34].

In conclusion, HTLV-1 was associated with sarcopenia in Japanese community dwellers. Similar to HIV infection, active surveillance and early detection of asymptomatic HTLV-1 infection might help reinforce countermeasures to prevent and inhibit the progress of HTLV-1 infection associated sarcopenia.

## MATERIALS AND METHODS

### Study settings and participants

We conducted this cross-sectional study using data from the Nagasaki Islands Study, which was a prospective cohort study performed in Goto City in the western islands of Japan [35]. Details of the selection process and procedures of the examination in this study were published previously [35, 36].

The participants were recruited at medical check-ups, and members of the general population aged ≥ 40 years living in Goto City were targeted for enrolment. The Ethical Committee of Nagasaki University approved this study (project registration number: 14051404, 20141002-5; Nagasaki, Japan), which was conducted according to the ethical standards defined in the 1964 Declaration of Helsinki as well as its subsequent amendments. Written informed consent was obtained from all participants. Of 3,365 participants enrolled from 2017 to 2019, 2,811 participants were included after exclusion for the following: age <40 years (n = 73), history of stroke (n = 155), BMI < 18.5 to avoid the effects of undernutrition on sarcopenia (n = 233), missing data for HTLV-1 serostatus (n = 14), handgrip strength (n = 7), ASM (n = 70), LDL (n = 1), and history of hypoglycaemic agent use (n = 1). Finally, 2,811 participants (1,066 men and 1,745 women) with a mean age of 70.2 years (SD, 10.0 years; range, 40–97 years) were evaluated.

### Data collection and laboratory measurements

Body weight and height were measured with participants wearing light-weight clothes and without shoes, and the BMI was then calculated. Body composition was analysed by multi-frequency bioelectrical impedance analysis using InBody 430 (InBody Japan, Tokyo, Japan). The muscle mass of the four limbs as ASM was summated. SMI (kg/m^2^) was calculated as the ASM (kg) divided by the square of the height (m) defined by Baumgartner’s formula [37]. Handgrip strength was recorded with the participant in a standing posture with his/her arm extended in a natural position. The handgrip dynamometer was adjusted for the participants so that their second proximal phalanxes were positioned around the handle. Handgrip strength was measured twice in both hands and the maximum scores of all recorded values for both sides were considered for analysis (Matsumiya Ika Seiki Seisakujo Smedley Dynamometer 0-1019-01).

SBP and diastolic blood pressure (DBP) at rest were recorded with a blood pressure measuring device (HEM-907; Omron, Kyoto, Japan). The measurements were repeated when the SBP was ≥ 140 mmHg or DBP was ≥ 90 mmHg, and the mean values were used for analyses. We used a questionnaire to obtain information regarding each participant’s medical history of stroke, use of antihypertensive agents, hypoglycaemic agents, and lipid-lowering drugs. Smoking status and drinking status were categorized as “never”, “ex”, or “current”. Physical activity was evaluated using two questions: “Do you have a daily walking habit?” and “Do you regularly exercise more than 30 minutes in a day? (Yes, No)”. Physical activity was scored as the total sum of “Yes” answers to the two questions.

Fasting blood samples were collected at the time of clinical examination. Blood samples were collected in an EDTA-2K tube, a siliconized tube, and a sodium fluoride tube. Samples from the EDTA-2K tube were used to measure the levels of WBC using an automated procedure at SRL, Inc. (Tokyo, Japan). Serum concentrations of LDL, HDL, TG, creatinine, HbA1c, and hs-CRP were measured by standard laboratory procedures.

### Measurements of HTLV-1

CLEIA kit (Fujirebio Inc., Tokyo, Japan) was used for HTLV-1 detection. As a confirmatory test, we used RT-PCR using the Hydrolysis probe method with the LightCycler 480 (Roshe, Basel, Schweiz) as previously reported [38]. Sample DNA was purified from whole blood using GENE PREP STAR NA-480 (KURABO, Osaka, Japan). In addition, we used western blotting (Problot HTLV-1, Fujirebio Inc., Tokyo, Japan) or Innogenetics line immunoassay (INNO-LIA HTLV, Fujirebio Inc., Tokyo, Japan) for the RT-PCR-negative cases.

### Definition of sarcopenia

Patients with a handgrip strength <26 kg for men and <18 kg for women, and SMI <7.0 kg/m^2^ for men and <5.7 kg/m^2^ for women were diagnosed as sarcopenia using Asian cutoff values [6, 39]. Low handgrip strength or low SMI were also defined using these cutoff values.

### Statistical analyses

Differences in mean values or proportions of variables by HTLV-1 positivity were analysed using the Wilcoxon rank sum test for continuous variables (age, height, weight, BMI, handgrip strength, ASM, physical activity, SBP, DBP, HbA1c, LDL, HDL, TG, hs-CRP and WBC), or the McNemar chi-square test for categorical variables (sex, sarcopenia, smoking status, drinking status, antihypertensive drug use, hypoglycemic drug use and lipid-lowering drug use). We performed simple linear regression analysis of handgrip strength and ASM as continuous variables. Then, we performed logistic regression analysis using clinical cutoff points of low handgrip strength, low SMI and sarcopenia. In the multivariable logistic regression analysis, adjustments were made *a priori* for age, BMI, SBP, HbA1c, LDL, physical activity (continuous variables), sex (dichotomous variable), and smoking and drinking status (categorical variable; never, ex, current: 1, 2, 3, respectively) [17, 35]. Age, sex, and BMI were included in adjustment Model 1. Age, sex, BMI, physical activity, SBP, HbA1c, LDL, and smoking and drinking status were included in Model 2. Because hs-CRP and WBC had a skewed distribution, they were expressed as the median and interquartile range, followed by logarithmic transformation.

Because markers of chronic inflammation may play an important role in sarcopenia progression [14], we also evaluated the association between sarcopenia and inflammation markers (hs-CRP and WBC). For sensitivity analyses, we performed analysis among participants who were excluded as premenopausal women (n = 84) because menopause is associated with a decline in oestrogen that decreases muscle mass and strength [40]. All P values for statistical tests were two-tailed and P < 0.05 was considered significant. All statistical analyses were performed using STATA v14 (StataCorp, College Station, TX, USA).

## References

[1] Cruz-Jentoft AJ, Sayer AA. Sarcopenia. Lancet. 2019; 393:2636–46. 10.1016/S0140-6736(19)31138-9 31171417

[2] Anker SD, Morley JE, von Haehling S. Welcome to the ICD-10 code for sarcopenia. J Cachexia Sarcopenia Muscle. 2016; 7:512–14. 10.1002/jcsm.12147 27891296 PMC5114626

[3] Rantanen T, Guralnik JM, Foley D, Masaki K, Leveille S, Curb JD, White L. Midlife hand grip strength as a predictor of old age disability. JAMA. 1999; 281:558–60. 10.1001/jama.281.6.558 10022113

[4] Rantanen T, Harris T, Leveille SG, Visser M, Foley D, Masaki K, Guralnik JM. Muscle strength and body mass index as long-term predictors of mortality in initially healthy men. J Gerontol A Biol Sci Med Sci. 2000; 55:M168–73. 10.1093/gerona/55.3.m168 10795731

[5] Al Snih S, Markides KS, Ottenbacher KJ, Raji MA. Hand grip strength and incident ADL disability in elderly Mexican Americans over a seven-year period. Aging Clin Exp Res. 2004; 16:481–86. 10.1007/BF03327406 15739601

[6] Cruz-Jentoft AJ, Bahat G, Bauer J, Boirie Y, Bruyère O, Cederholm T, Cooper C, Landi F, Rolland Y, Sayer AA, Schneider SM, Sieber CC, Topinkova E, et al, and Writing Group for the European Working Group on Sarcopenia in Older People 2 (EWGSOP2), and the Extended Group for EWGSOP2. Sarcopenia: revised European consensus on definition and diagnosis. Age Ageing. 2019; 48:16–31. 10.1093/ageing/afy169 30312372 PMC6322506

[7] Goates S, Du K, Arensberg MB, Gaillard T, Guralnik J, Pereira SL. Economic impact of hospitalizations in US adults with sarcopenia. J Frailty Aging. 2019; 8:93–99. 10.14283/jfa.2019.1030997923

[8] Cleasby ME, Jamieson PM, Atherton PJ. Insulin resistance and sarcopenia: mechanistic links between common co-morbidities. J Endocrinol. 2016; 229:R67–81. 10.1530/JOE-15-0533 26931135

[9] Marzetti E, Calvani R, Cesari M, Buford TW, Lorenzi M, Behnke BJ, Leeuwenburgh C. Mitochondrial dysfunction and sarcopenia of aging: from signaling pathways to clinical trials. Int J Biochem Cell Biol. 2013; 45:2288–301. 10.1016/j.biocel.2013.06.024 23845738 PMC3759621

[10] Renoud A, Ecochard R, Marchand F, Chapurlat R, Szulc P. Predictive parameters of accelerated muscle loss in men-MINOS study. Am J Med. 2014; 127:554–61. 10.1016/j.amjmed.2014.02.004 24524994

[11] Lee JS, Auyeung TW, Kwok T, Lau EM, Leung PC, Woo J. Associated factors and health impact of sarcopenia in older Chinese men and women: a cross-sectional study. Gerontology. 2007; 53:404–10. 10.1159/000107355 17700027

[12] Seo JA, Cho H, Eun CR, Yoo HJ, Kim SG, Choi KM, Baik SH, Choi DS, Park MH, Han C, Kim NH. Association between visceral obesity and sarcopenia and vitamin D deficiency in older Koreans: the ansan geriatric study. J Am Geriatr Soc. 2012; 60:700–06. 10.1111/j.1532-5415.2012.03887.x 22316299

[13] Lord C, Chaput JP, Aubertin-Leheudre M, Labonté M, Dionne IJ. Dietary animal protein intake: association with muscle mass index in older women. J Nutr Health Aging. 2007; 11:383–87. 17657359

[14] Visser M, Pahor M, Taaffe DR, Goodpaster BH, Simonsick EM, Newman AB, Nevitt M, Harris TB. Relationship of interleukin-6 and tumor necrosis factor-alpha with muscle mass and muscle strength in elderly men and women: the health ABC study. J Gerontol A Biol Sci Med Sci. 2002; 57:M326–32. 10.1093/gerona/57.5.m326 11983728

[15] Cesari M, Kritchevsky SB, Baumgartner RN, Atkinson HH, Penninx BW, Lenchik L, Palla SL, Ambrosius WT, Tracy RP, Pahor M. Sarcopenia, obesity, and inflammation—results from the trial of angiotensin converting enzyme inhibition and novel cardiovascular risk factors study. Am J Clin Nutr. 2005; 82:428–34. 10.1093/ajcn.82.2.428 16087989

[16] Roubenoff R. Sarcopenia: effects on body composition and function. J Gerontol A Biol Sci Med Sci. 2003; 58:1012–17. 10.1093/gerona/58.11.m1012 14630883

[17] Yamanashi H, Kulkarni B, Edwards T, Kinra S, Koyamatsu J, Nagayoshi M, Shimizu Y, Maeda T, Cox SE. Association between atherosclerosis and handgrip strength in non-hypertensive populations in India and Japan. Geriatr Gerontol Int. 2018; 18:1071–78. 10.1111/ggi.13312 29582539 PMC6144064

[18] Hawkins KL, Brown TT, Margolick JB, Erlandson KM. Geriatric syndromes: new frontiers in HIV and sarcopenia. AIDS. 2017 (Suppl 2); 31:S137–46. 10.1097/QAD.0000000000001444 28471944 PMC5693390

[19] Abdul Aziz SA, Mcstea M, Ahmad Bashah NS, Chong ML, Ponnampalavanar S, Syed Omar SF, Sulaiman H, Azwa I, Tan MP, Kamarulzaman A, Rajasuriar R, Kamaruzzaman SB. Assessment of sarcopenia in virally suppressed HIV-infected Asians receiving treatment. AIDS. 2018; 32:1025–34. 10.1097/QAD.0000000000001798 29547442

[20] Proietti FA, Carneiro-Proietti AB, Catalan-Soares BC, Murphy EL. Global epidemiology of HTLV-I infection and associated diseases. Oncogene. 2005; 24:6058–68. 10.1038/sj.onc.1208968 16155612

[21] Morgan OS, Rodgers-Johnson P, Mora C, Char G. HTLV-1 and polymyositis in Jamaica. Lancet. 1989; 2:1184–87. 10.1016/s0140-6736(89)91793-5 2572904

[22] Desdouits M, Cassar O, Maisonobe T, Desrames A, Aouba A, Hermine O, Mikol J, Polivka M, Penisson-Besnier I, Marcorelles P, Zagnoli F, Papo T, Lacour A, et al. HTLV-1-associated inflammatory myopathies: low proviral load and moderate inflammation in 13 patients from West Indies and West Africa. J Clin Virol. 2013; 57:70–76. 10.1016/j.jcv.2012.12.016 23375238

[23] Schrack JA, Althoff KN, Jacobson LP, Erlandson KM, Jamieson BD, Koletar SL, Phair J, Ferrucci L, Brown TT, Margolick JB, and Multicenter AIDS Cohort Study. Accelerated longitudinal gait speed decline in HIV-infected older men. J Acquir Immune Defic Syndr. 2015; 70:370–76. 10.1097/QAI.0000000000000731 26102450 PMC4624470

[24] Schrack JA, Jacobson LP, Althoff KN, Erlandson KM, Jamieson BD, Koletar SL, Phair J, Brown TT, Margolick JB, and Multicenter AIDS Cohort Study. Effect of HIV-infection and cumulative viral load on age-related decline in grip strength. AIDS. 2016; 30:2645–52. 10.1097/QAD.0000000000001245 27603294 PMC5083134

[25] Futsch N, Prates G, Mahieux R, Casseb J, Dutartre H. Cytokine networks dysregulation during HTLV-1 infection and associated diseases. Viruses. 2018; 10:691. 10.3390/v10120691 30563084 PMC6315340

[26] Zhao LJ, Giam CZ. Human t-cell lymphotropic virus type I (HTLV-I) transcriptional activator, tax, enhances CREB binding to HTLV-I 21-base-pair repeats by protein-protein interaction. Proc Natl Acad Sci USA. 1992; 89:7070–74. 10.1073/pnas.89.15.7070 1386673 PMC49647

[27] Haran PH, Rivas DA, Fielding RA. Role and potential mechanisms of anabolic resistance in sarcopenia. J Cachexia Sarcopenia Muscle. 2012; 3:157–62. 10.1007/s13539-012-0068-4 22589021 PMC3424190

[28] Costamagna D, Costelli P, Sampaolesi M, Penna F. Role of inflammation in muscle homeostasis and myogenesis. Mediators Inflamm. 2015; 2015:805172. 10.1155/2015/805172 26508819 PMC4609834

[29] Kinjo T, Ham-Terhune J, Peloponese JM Jr, Jeang KT. Induction of reactive oxygen species by human t-cell leukemia virus type 1 tax correlates with DNA damage and expression of cellular senescence marker. J Virol. 2010; 84:5431–37. 10.1128/JVI.02460-09 20219913 PMC2863840

[30] Takahashi M, Higuchi M, Makokha GN, Matsuki H, Yoshita M, Tanaka Y, Fujii M. HTLV-1 tax oncoprotein stimulates ROS production and apoptosis in T cells by interacting with USP10. Blood. 2013; 122:715–25. 10.1182/blood-2013-03-493718 23775713

[31] Fulle S, Protasi F, Di Tano G, Pietrangelo T, Beltramin A, Boncompagni S, Vecchiet L, Fanò G. The contribution of reactive oxygen species to sarcopenia and muscle ageing. Exp Gerontol. 2004; 39:17–24. 10.1016/j.exger.2003.09.012 14724060

[32] Ferrucci L, de Cabo R, Knuth ND, Studenski S. Of greek heroes, wiggling worms, mighty mice, and old body builders. J Gerontol A Biol Sci Med Sci. 2012; 67:13–16. 10.1093/gerona/glr046 22113943 PMC3260484

[33] Nagai M, Usuku K, Matsumoto W, Kodama D, Takenouchi N, Moritoyo T, Hashiguchi S, Ichinose M, Bangham CR, Izumo S, Osame M. Analysis of HTLV-I proviral load in 202 HAM/TSP patients and 243 asymptomatic HTLV-I carriers: high proviral load strongly predisposes to HAM/TSP. J Neurovirol. 1998; 4:586–93. 10.3109/13550289809114225 10065900

[34] Gilbert DT, Morgan O, Smikle MF, Simeon D, Barton EN. HTLV-1 associated polymyositis in Jamaica. Acta Neurol Scand. 2001; 104:101–04. 10.1034/j.1600-0404.2001.104002101.x 11493227

[35] Yamanashi H, Koyamatsu J, Nagayoshi M, Shimizu Y, Kawashiri SY, Kondo H, Fukui S, Tamai M, Sato S, Yanagihara K, Kawakami A, Maeda T. Human t-cell leukemia virus-1 infection is associated with atherosclerosis as measured by carotid intima-media thickness in Japanese community-dwelling older people. Clin Infect Dis. 2018; 67:291–94. 10.1093/cid/ciy168 29529133

[36] Yamanashi H, Shimizu Y, Koyamatsu J, Nobuyoshi M, Nagayoshi M, Kadota K, Tamai M, Maeda T. Multiple somatic symptoms and frailty: cross-sectional study in Japanese community-dwelling elderly people. Fam Pract. 2016; 33:453–60. 10.1093/fampra/cmw028 27130337

[37] Baumgartner RN, Koehler KM, Gallagher D, Romero L, Heymsfield SB, Ross RR, Garry PJ, Lindeman RD. Epidemiology of sarcopenia among the elderly in new Mexico. Am J Epidemiol. 1998; 147:755–63. 10.1093/oxfordjournals.aje.a009520 9554417

[38] Chen LK, Liu LK, Woo J, Assantachai P, Auyeung TW, Bahyah KS, Chou MY, Chen LY, Hsu PS, Krairit O, Lee JS, Lee WJ, Lee Y, et al. Sarcopenia in Asia: consensus report of the Asian working group for sarcopenia. J Am Med Dir Assoc. 2014; 15:95–101. 10.1016/j.jamda.2013.11.025 24461239

[39] Cassar O, Gessain A. Serological and molecular methods to study epidemiological aspects of human t-cell lymphotropic virus type 1 infection. Methods Mol Biol. 2017; 1582:3–24. 10.1007/978-1-4939-6872-5_1 28357658

[40] Maltais ML, Desroches J, Dionne IJ. Changes in muscle mass and strength after menopause. J Musculoskelet Neuronal Interact. 2009; 9:186–97. 19949277

